# The Duration of Impella 2.5 Circulatory Support and Length of Hospital Stay of Patients Undergoing High-risk Percutaneous Coronary Interventions

**DOI:** 10.4021/cr190e

**Published:** 2012-07-20

**Authors:** Obiora Anusionwu, Daniel Fischman, Pramil Cheriyath

**Affiliations:** a Department of Hospital Medicine, Johns Hopkins University School of Medicine, Baltimore, Maryland 21224, United States of America; b Department of Internal Medicine, Pinnacle Health Systems, Harrisburg, Pennsylvania 17104, United States of America

**Keywords:** Atherosclerosis, Left ventricular assist devices, Myocardial infarction, Percutaneous coronary interventions

## Abstract

**Background:**

To evaluate the impact of duration of Impella 2.5 support (Abiomed, Danvers, MA) on hospitalization of patients after high-risk percutaneous coronary intervention (PCI). There has been a continuous increase in prevalence of coronary artery disease with more patients needing PCI during acute myocardial infarction. Some of these patients have to undergo high-risk revascularization with circulatory support like the Impella 2.5 device.

**Methods:**

This study was a single center retrospective study of patients admitted to our hospital who required Impella circulatory support during percutaneous coronary intervention. Patients’ medical records, cardiac catheterization laboratory and 2-D echocardiography reports were reviewed to ascertain left ventricular ejection fraction, duration of Impella support, Coronary Care Unit (CCU) days and the length of stay in the hospital. A P-value of ≤ 0.05 was considered statistically significant.

**Results:**

Over a 15-month period, we had 25 patients with 19 males and 6 females. Mean age of the patient cohort was 68 ± 10 years. Mean LVEF of the group was 32 ± 16%. Mean length of hospital stay was 8 ± 8 days and mean CCU stay was 4 ± 4 days. The Impella was successfully inserted in all cases with a median duration of support of 70 minutes (range, 4 - 5760 minutes). Bleeding complication occurred in 8%. Spearman's rank correlation coefficient between the duration of Impella support and hospital stay was 0.49 (P = 0.023) while it was 0.71 (P = 0.001) between Impella support duration and CCU days.

**Conclusions:**

Our study suggests that there is a positive correlation between the duration of Impella 2.5 circulatory support and hospital stay and/or CCU days. The correlation seems to be stronger with CCU days.

## Introduction

The prevalence of cardiovascular disease (CVD) is increasing and would continue to be the leading cause of death and disability in the world by the 2020 according to the World Health Organization estimates [1]. Several innovative research activities have been geared towards decreasing its prevalence. Coronary artery disease is one of the commonest causes of CVD and remains one of the leading causes of death in the United States [2]. Recently, left ventricular assist devices (LVAD) have been used to manage the patients with coronary artery disease who are undergoing elective or emergent percutaneous coronary interventions (PCI). One of such LVAD is the Impella 2.5 device (Abiomed, Danvers, MA, U.S.A.), which is used to percutaneously support patient’s circulatory system while undergoing high-risk percutaneous coronary interventions (PCI). Patients presenting in cardiogenic shock can also benefit from the hemodynamic support of the device by the improvement of their mean arterial pressure and cardiac index especially if they require PCI for revascularization [3].

The Impella 2.5 LVAD consists of a small microaxial pump that is usually inserted in a retrograde manner percutaneously in the femoral artery and, after it is advanced into the heart, sits in the left ventricle [4]. It is able to pump out blood and unload the contents of the left ventricle into the ascending aorta via the rotary miniaturized microaxial pump hence improving cardiac output, circulatory support and coronary blood flow. It is able to achieve about 2.5 L/min of blood flow per cardiac output. The United States Food and Drug Administration approved this device in June 2008 for up to 6 hours of support [5]. However, in Europe, the device is approved for up to 5 days of partial circulatory support [5].

Several studies have been carried out on the feasibility and safety of the Impella device [4, 6-8] but there is insufficient data on the effect of the duration of support with the Impella device on the length of the hospitalization of patients. Hence we hypothesized that the patients who require longer support on the Impella 2.5 device during their high-risk PCI would have a longer hospital and coronary care unit stay.

## Materials and Methods

### Patient population

In this retrospective study, we enrolled all the consecutive patients that were admitted to our hospital from May 2008 to July 2009 and received the Impella 2.5 during their high- risk PCI. These patients were identified from the medical registry. The decision to use the Impella 2.5 during the PCI was determined by the Interventional Cardiologist, and is based on the patient’s presentation and medical history. Four patients (all male) did not have available information about Impella support length because it was absent in their medical records.

### Study protocol

The Institutional Review Board of our hospital approved the research protocol. Patients’ charts, coronary catheterization reports, laboratory results, transthoracic echocardiogram (TTE) reports and discharge summaries were reviewed to identify the age, gender, left ventricular ejection fraction and the duration that the Impella support was used. Hospitalization was measured using entire hospital stay and coronary care unit (CCU) stay.

### Procedure details

The patients were admitted to the hospital because they required high-risk PCI using the Impella 2.5. The device was inserted percutaneously through the femoral artery using a 13Fr femoral catheter sheath and goes in a retrograde fashion into the left ventricle. Its tip has a microaxial pump that is able to pump about 2.5 L/min of blood for circulatory support during the procedure. TTE is usually done before insertion of the Impella to rule out some of the contraindications of implantation like mechanical prosthetic aortic valve, severe aortic stenosis and left ventricular thrombus. While the Impella is in place, the patients were placed on anticoagulation using heparin. Dixon et al describes further details on the procedure for Impella insertion in Protect I trial [6].

### Statistical analysis

Continuous variables are expressed as mean ± SD or median (range). Categorical variables are expressed as frequency (percentage). Spearman’s nonparametric correlation was used for analysis of the continuous variables using STATA statistical analysis software (STATA Corp v11.2, 2011, College Station, TX). The Spearman rho was used to analyze the correlation (magnitude and direction of the association) between Impella support duration and hospital and/or CCU stay. Plots were also prepared to review the association between the variables. A P-value of ≤ 0.05 was considered statistically significant.

## Results

The Impella 2.5 was successfully implanted and explanted in all 25 patients identified from the medical registry. The mean age of the cohort was 68 years ± 9.5. There were nineteen males and six females. Table 1 illustrates the characteristics of the patient which are as follows: 60% were smokers, 92% had hypertension, 72% had diabetes, 84% had hyperlipidemia, 36% had prior coronary artery bypass graft surgery, 32% had chronic kidney disease, 76% had previous myocardial infarction, 28% had previous percutaneous coronary interventions and 8% had automated implantable cardioverter-defibrillator. The mean left ventricular ejection fraction of the patient cohort was 32 ± 16% (median = 35%, range: 10-70%). Mean length of hospital stay was 8 ± 8 days (median = 5, range: 2 - 39 days). Mean CCU stay was 4 ± 4 days (median = 2, range: 1 - 17 days). The duration of Impella support ranged from 4 to 5740 minutes with a median of 70 minutes and mean of 603 ± 1523 minutes.

**Table 1:** Patient Characteristics (n = 25)

Patient Characteristics	Mean ± SD	N (%)
Age (years)	68 ± 9.5	
LV Ejection fraction (%)	35 ± 16.4	
Current smokers		15 (60)
Hypertension		23 (92)
Diabetes mellitus		18 (72)
Hyperlipidemia		21 (84)
Prior CABG		10 (36)
Chronic kidney disease		8 (32)
Previous MI		19 (76)
Previous PCI		7 (28)
Has AICD		2 (8)

CABG: coronary artery bypass graft; MI: myocardial infarction; PCI: percutaneous coronary intervention; AICD: automatic implantable cardioverter defibrillator; LV: left ventricle.

Statistical analysis was done using the Spearman’s nonparametric correlation. For the 21 patients whose data could be analyzed, the Spearman’s rank correlation coefficient (rho) was 0.49 for hospital days and the duration of Impella support (P = 0.023). Also, the Spearman’s rho for CCU days and Impella support duration was 0.71 (P < 0.001).

### Bleeding complication

Patients were placed on heparin during the time the Impella 2.5 device was in place. Bleeding complication in the form of groin hematoma was noticed in 8% (n = 2) of patients. This was treated with manual compression and it resolved.

## Discussion

This is an important study that looks at a group of patients requiring left ventricular assist device support for high-risk PCI and correlating it with their hospitalization. Our study emphasizes the importance of expecting a longer coronary care unit stay and hospital stay for patients presenting for high-risk PCI and requiring Impella circulatory support. In a recent paper, high-risk PCI was described as left main intervention with EF < 35%, intervention with 3 vessel disease in patients with EF < 30% or intervention on the last patent coronary conduit [9]. Our results showed there was a statistically significant positive correlation between the duration of support on the Impella 2.5 device and CCU stay. There was also a statistically significant positive correlation between the duration of support of the Impella 2.5 device and the length of hospital stay. The Spearman’s rho coefficient for CCU stay was higher than hospital stay which is interesting since patients usually go to the CCU after PCI. This is important because longer hospital and coronary care unit stays could be associated with other complications and co-morbidities of which the patient and their family have to be aware off. This adds to the body of medical literature on circulatory support devices and its correlation with hospitalization and patient outcomes.

Furthermore, this study emphasized the capability of implanting and explanting the Impella 2.5 device since this was achieved in all of our patients. The patients in our study had decreased cardiac function as evidenced by the mean left ventricular ejection fraction of 32 ± 16%. This is in accordance with a previous study where the device was successfully placed in all patients with no major complication from the Impella device [4]. Most of our patients before undergoing the high-risk PCI had been deemed unfit for CABG based on their present and past history, multiple comorbidities and physician evaluations. Some of the patients already had CABG, previous MI, AICD or previous PCI. In our study 2 patients had bleeding complications while in Henriques et al, they had a patient that bled and required blood transfusion [4]. In the study by Seyfarth et al, higher hemolysis rates and more blood products were administered to patients treated with Impella [3]. This bleeding complication could be because the device is in the large arteries and patients are usually on anticoagulation to help prevent clot formation.

Finally, there have been increased interest on the safety and feasibility of the use of Impella 2.5 device in the ‘real word setting’ but several studies have demonstrated its capability in this regard [4, 6-8]. In the US, several trials like Protect I, Protect II, ISAR-SHOCK, Recover I and Recover II trials have been sponsored by ABIOMED to help answer this questions with encouraging results [5]. It has also been shown that it can help improve coronary blood flow and cardiac output by reducing the workload and oxygen consumption of the cardiac muscle [10-12]. In this study, the device was implanted and explanted safely in all the patients with minimal bleeding complication. This is similar to a recent study, which showed that their patients experienced a high rate of procedural success and a reasonable length of stay post-intervention with a low 30-day MACE rate at follow-up [13].

### Limitations

Some the limitations of our study includes difficulty in retrieving information from patients’ records. In addition, this was a single center study with a small sample size in a predominately male population. Finally, our study inherently has all the limitations that have been observed in retrospective study designs.

### Conclusion

The Impella 2.5 is a feasible and relatively safe circulatory support device that can be used for high-risk PCI. Longer duration of support on the Impella 2.5 device correlates more strongly with longer CCU stays and less with hospital days.
